# Exploring antiemetic strategies in hematologic malignancies: a comprehensive literature review and evaluation of antiemetic efficacy in patients receiving high-dose chemotherapy prior to hematopoietic stem cell transplantation

**DOI:** 10.1007/s00520-025-09638-9

**Published:** 2025-06-24

**Authors:** Karin Jordan, Berit Jordan, Jennifer Vanden Burgt, Franziska Jahn

**Affiliations:** 1 Department of Hematology, Oncology and Palliative Care, Ernst Von Bergmann Hospital, Potsdam, Germany; 2 Department of Hematology, Oncology and Rheumatology, University Hospital Heidelberg, Heidelberg, Germany; 3 Department of Neurology, Martin Luther University Halle-Wittenberg, Halle, Germany; 4 Independent Consultant, Medical Affairs, Oncology , Minneapolis, MN USA; 5 Department of Internal Medicine IV, Hematology/Oncology, Martin Luther University Halle-Wittenberg, Halle, Germany

**Keywords:** Antiemetics, CINV, Nausea, Vomiting, Hematology, Transplantation

## Abstract

**Purpose:**

Most antiemetic studies have been conducted in patients with solid tumors receiving single-dose chemotherapy. A research gap leaves healthcare providers without clear guidance on effective antiemetic regimens and schedules for patients with hematologic malignancies undergoing high-dose multiday chemotherapy. This literature search identified antiemetic studies and assessed efficacy outcomes in the hematology setting, and specifically in patients receiving high-dose chemotherapy prior to hematopoietic stem cell transplantation (HSCT).

**Methods:**

A literature review of both PubMed and Embase was performed for published studies evaluating antiemetic regimens including an NK1 receptor antagonist (RA) and/or a 5HT-3RA with/without dexamethasone in the hematology setting. Key features of all studies are reviewed, and antiemetic efficacy is summarized specifically for studies in which patients received high-dose chemotherapy prior to HSCT.

**Results:**

Twenty-two of an initial 926 identified publications met the predefined inclusion criteria. The studies were heterogenous, with varying characteristics pertaining to randomization, control groups, size, cancer types, chemotherapies, antiemetics, and assessments, making cross-study comparisons and conclusions difficult. The range of response rates was wide with numerous studies showing complete response, no emesis or no nausea rates of less than 50%. Response rates were highest when an NK1 RA regimen was administered; however, an NK1 RA was underutilized and only administered in two-thirds of the studies.

**Conclusion:**

The results reflect a significant clinical problem in preventing chemotherapy-induced nausea and vomiting (CINV) in patients with hematologic malignancies. The scarcity and heterogeneity of studies highlight challenges inherent in this area. This underscores a pressing need for rigorous randomized trials in hematology and HSCT assessing treatment-related CINV and effectiveness of antiemetic regimens.

## Introduction

Nausea and vomiting are common side effects of many chemotherapeutic agents which can have a deleterious impact on patients’ quality of life (QoL) and may result in chemotherapy dose reductions or discontinuation [1]. Fortunately, the past four decades of research have brought forth a variety of effective antiemetics, transforming the presence of these debilitating symptoms for patients. Navari and Aapro published a comprehensive overview of the history of antiemetics [2], highlighting that the American Society of Clinical Oncology (ASCO) has recognized the progress in this field as one of the top 5 advances in 50 years of modern oncology [3].

Concurrent with the ongoing development of new and effective antiemetics, classifications of the emetogenicity of antineoplastic agents were established and continue to evolve, enabling antiemetic regimens to be tailored and refined for patients based on the emetogenic potential of the chemotherapy [4, 5]. Similarly, national and international antiemetic guidelines were developed in the late 1990s to guide health care providers on the optimal use of antiemetic agents [6–8]. These guidelines are now regularly revisited and revised as new agents become available [9–12]. allowing for effective prevention of chemotherapy-induced nausea and vomiting (CINV) for most patients if guidelines are followed [13, 14].

The preponderance of antiemetic studies, particularly those required for registration purposes of individual agents, has been conducted in patients with solid tumors receiving single-dose chemotherapy. Therefore, a gap exists in the research and consequently, in the guidelines, for directing healthcare providers on the optimal antiemetics and regimens in patients with hematologic malignancies receiving high-dose chemotherapy which usually corresponds with high emetogenic potential.

Managing CINV in the hematopoietic stem cell transplant (HSCT) and hematologic malignancy setting presents unique challenges. In acute myeloid leukemia induction, for example, dexamethasone is often omitted due to infection-related risks, while steroid use in allogeneic conditioning—especially with post-transplant cyclophosphamide (PtCy) regimens—may be limited because of their immunomodulatory effects. The concomitant use of cyclosporin and steroids further complicates care by inducing hyperglycemia and additive toxicity. Additionally, potential drug interactions between NK1 receptor antagonists (RAs) and agents such as cyclosporin and busulfan add another layer of complexity. Prevention of CINV is furthermore challenging for patients receiving high-dose chemotherapy regimens before HSCT due to the daily and continuous emetogenic stimulus of the multiple day chemotherapy. These factors, combined with the fact that patients are not chemotherapy-naïve and may experience anticipatory emesis, underscore the difficulties in designing and conducting robust randomized controlled trials in this population.

While guidelines generally suggest that adult patients who are treated with high-dose chemotherapy and HSCT should be offered a three- or four-drug combinations of a 5-HT3 RA, an NK1 RA, and dexamethasone (DEX) plus or minus olanzapine [9, 10, 15], detailed and specific recommendations are not offered on how to optimally administer the combination antiemetic regimen for the various conditioning regimens in the HSCT setting, where the doses and schedules of chemotherapy are usually more complicated than in the solid tumor setting. Similarly, there is no guideline consensus on the emetogenicity of these conditioning regimens.

A recent survey was conducted with 348 hematologists/oncologists in Europe to explore their perspectives on antiemetic use and CINV prevalence in patients undergoing HSCT for hematologic malignancies as well as to identify areas of unmet need in this setting [16]. Key learnings of this survey were that the majority of respondents indicated that a substantial proportion of their patients undergoing HSCT experienced CINV severe enough to impact functioning/QoL and that NK1 RA-containing regimens are considerably underutilized as antiemetic prophylaxis in patients receiving high-dose chemotherapy conditioning regimens prior to HSCT.

As a follow-up to this survey and to further explore the available evidence in the absence of specific guideline recommendations, a literature search was conducted to identify antiemetic studies in the hematology setting. Herein, we summarize the key features of the studies identified and highlight the efficacy outcomes in the HSCT setting.

## Methods

### Literature search and strategy

Review of both PubMed and Embase was performed for publications between 1 January 2003 (just prior to the approval of the first NK1 RA, aprepitant) and 1 May 2024, utilizing the following search terms.

1. Cancer AND Hematology
2. Nausea OR vomiting
3. “Antiemetic” OR “NK1RA” OR “5HT-3RA”
4. Dexamethasone
5. Randomized controlled trial OR controlled clinical trial OR random* OR placebo OR clinical trials as topic [mesh] OR meta-analysis OR open label* OR practice guideline OR guideline

The search terms matched those used by the American Society of Clinical Oncology (ASCO) in their systematic literature review for updating their antiemetic guidelines [9]. The initial results reflect a combination of 1 and 2 and (3 or 4) and 5.

Per the fifth search criteria, the studies not only reflected randomized controlled trials but also other types of studies such as open-label observational trials.

Inclusion criteria included the following:

1. Hematologic cancer population of adults ≥ 18 years of age
2. Sample size of the study > 20 patients
3. Must assess prevention of CINV
4. Prospective study; not case reports, retrospective studies, or narrative reviews
5. Published in English

Included studies reflect those conducted primarily in patients with hematologic malignancies but may have also included subsets of patients with other cancers. Patients were receiving high-dose chemotherapy, which was defined as chemotherapy administered at significantly higher doses than standard regimens as part of conditioning before HSCT.

Retrospectively, studies conducted in the non-transplant setting were excluded to focus solely on HSCT studies. Outcomes of interest were assessments of prevention of CINV.

### Selection of relevant studies and data collection process

All titles and abstracts of the references were screened by Excerpta Medica. Publications were reviewed independently by Excerpta Medical and by Dr. Karin Jordan, for topic relevance, hematologic cancers, antiemetic treatments, study type, and other factors until a final selection reflected studies conducted in a hematology setting which evaluated antiemetic regimens including an NK1 RA and/or a 5HT-3RA with or without dexamethasone. A review of the full text was performed as needed for assessment of more complete details. Discrepancies about study inclusion or data extraction were resolved with further review and discussion until consensus was reached.

After reading and analyzing the selected articles, we extracted the following information: title, author(s), year of publication, methodology, study population, cancer types, chemotherapy, and antiemetics administered. Antiemetic efficacy was summarized as reported in the papers.

## Results

### Studies included

**Fig. 1:**
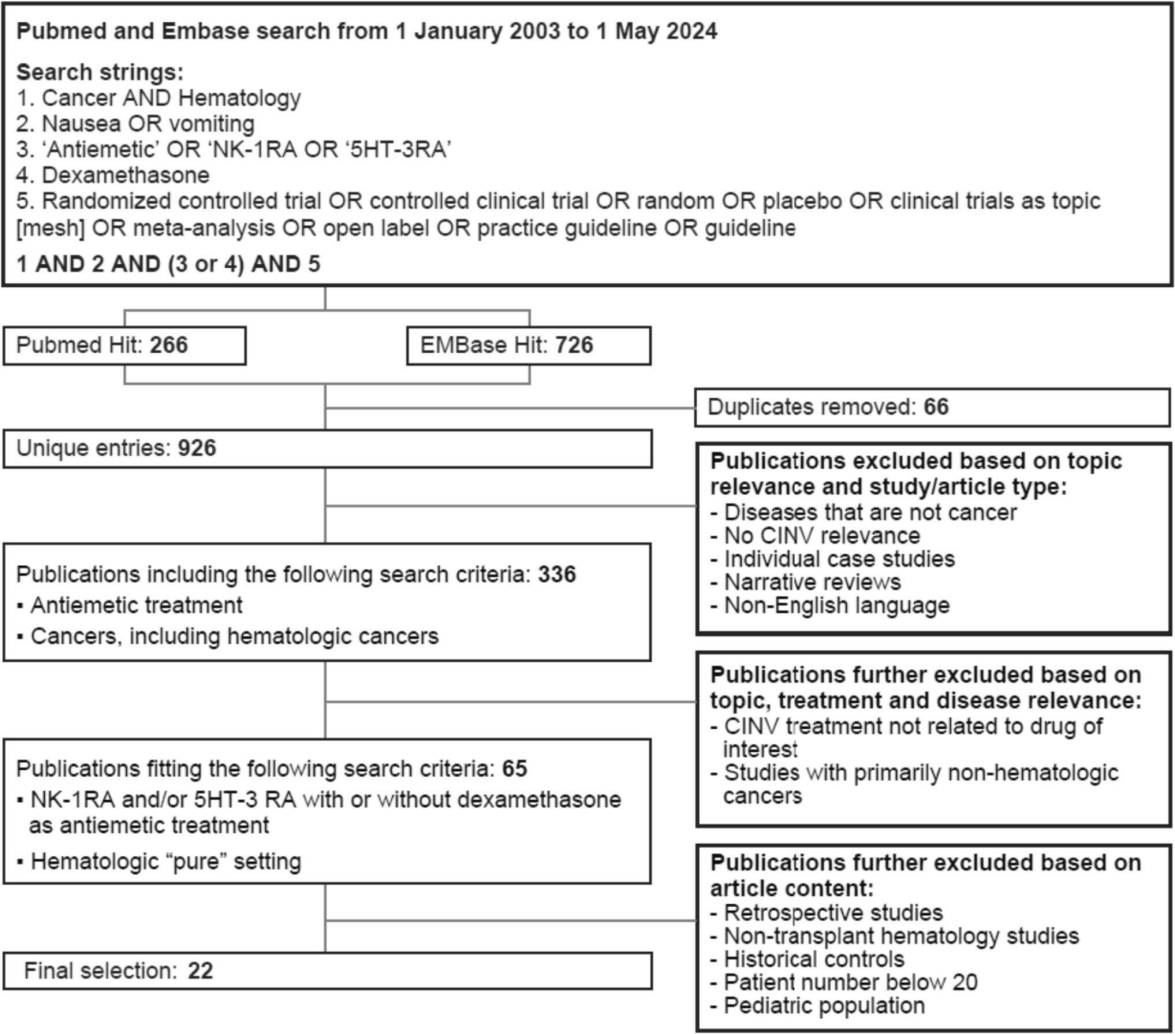
Flow diagram of included studies

Across the two databases, initial results included 926 unique publications (Fig. 1) of which 22 were identified as having met the predefined inclusion criteria. After exclusion of “non-transplant studies in the hematology setting, the final group of studies reflect those assessing antiemetics for preventing CINV during conditioning regimens received prior to HSCT (Table 1).

**Table 1:** Study design features

Publication	Study type	*N*	Type of HSCT	Conditioning regimen/chemotherapy	Primary cancer types	Primary antiemetic class(es)	Antiemetic study arms	Antiemetic regimens
**Allogenic**								
Yeh SP et al., 2014 [17]	SC, SA, OL	27	Allogenic	MAC (FluBu4) (*n* = 16) MAC (TBI 12 Gy + cyclophosphamide) (*n* = 1) RIC (FluBu2) (*n* = 5) RIC (FluCy) (*n* = 2) RIC (FluBu2Cy) (*n* = 3)	Acute leukemia (*n* = 20) CML (*n* = 2) MDS (*n* = 1) Severe aplastic anemia (*n* = 3) NHL (*n* = 1)	5-HT3 RA	PALO + DEX	Palonosetron 0.25 mg iv infusion, 30 min before the start of conditioning chemoradiotherapy and every other day through period of conditioning + DEX 10–15 mg iv daily during conditioning
**Autologous or both**								
NK1 RA + 5HT3 RA + DEX vs. 5-HT3 RA + DEX comparative studies								
**Predominantly melphalan chemotherapy regimens**								
Schmitt T et al., 2014 [18]	R, DB, SC	362	Autologous	Melphalan (*n* = 362) (100 mg/m^2^ days 1 and 2; AHSCT day 4)	MM	NK1 RA + 5-HT3 RA vs. 5-HT3 RA	APR + GRAN + DEX (*n* = 181) vs. GRAN + DEX (*n* = 181)	Aprepitant 125 mg on day 1, then 80 mg on days 2–4 + Granisetron 2 mg on days 1–4 + DEX 4 mg on day 1 and then 2 mg on days 2 and 3 vs. placebo + Granisetron 2 mg on days 1–4 + DEX 8 mg on day 1 and then 4 mg on days 2 and 3
Svanberg A et al., 2015 [19]	R, DB, SC	90	Autologous	Myeloma: high-dose melphalan (*n* = 51) (200 mg/m^2^ on 1 occasion for 1 single day) + BBM myeloma 2:HSCT (*n* = 2) Lymphoma: BEAC (*n* = 33) (carmustine 300 mg/m^2^ on day 1, etoposide 100 mg/m^2^ on 8 occasions during 5 days, cytarabine 100 mg/m^2^ on 8 occasions during 4 days, cyclophosphamide 35 mg/kg on 4 occasions during 4 days) or BEAM (*n* = 4) (carmustine 300 mg/m^2^ on day 1, etoposide 200 mg/m^2^ on 4 occasions during 4 days, cytarabine 200 mg/m^2^ on 8 occasions during 4 days, melphalan 140 mg/m^2^ on day 6)	Myeloma (*n* = 54) Lymphoma (*n* = 36)	NK1 RA + 5-HT3 RA vs. 5-HT3 RA	APR + Tropisetron + Betamethasone (*n* = 46) vs. Tropisetron + Betamethasone (*n* = 44)	Aprepitant + Tropisetron 5 mg + Betamethasone 6 mg 1 h before the 1st HDCT dose and daily until 7 days after the end of CT vs. Placebo + Tropisetron 5 mg + Betamethasone 6 mg 1 h before the 1st HDCT dose and daily until 7 days after the end of CT
**Cyclophosphamide chemotherapy regimens**								
Stiff PJ et al., 2013 [20]	R, SC, DB	179	Either auto (*n* = 89) allo (*n* = 90)	High-dose cyclophosphamide preparative regimens: TBI/Cy (*n* = 79) (TBI ¼ 1200 cGy fractionated into 8 doses on days − 7, − 6, − 5, and − 4, and Cy 60 mg/kg iv over 1 h days − 3 and − 2) IV Bu/Cy (*n* = 14) (Bu 0.8 mg/kg/dose every 6 h × 16 doses given on days − 7, − 6, − 5, − 4 and Cy 60 mg/kg iv over 1 h on days − 3 and − 2) Oral Bu/CY (*n* = 34) (Bu 0.875 mg/kg/dose, Cy 60 mg/kg iv over 1 h on days − 3 and − 2) TBI/VP/Cy (*N* = 36) (TBI 1200 Gy fractionated into 8 doses on days − 8, − 7, − 6, and − 5, etoposide 60 mg/kg iv over 4 h on day − 4, Cy 100 mg/kg iv over 2 h on day − 2) BCV (*N* = 16) (BCNU, etoposide, Cy (carmustine 15 mg/kg iv over 2 h on day − 6, etoposide 60 mg/kg iv over 4 h on day − 4, Cy 100 mg/kg iv over 2 h on day − 2)	NHL (*n* = 57) AML (*n* = 46) MM (*n* = 34) ALL (*n* = 13) HL (*n* = 12) CML (*n* = 6) Other (*n* = 11)	NK1 RA + 5-HT3 RA + DEX vs. 5-HT3 RA + DEX	APR + OND + DEX (*n* = 90) vs. OND + DEX (*n* = 89)	Aprepitant 125 mg orally on day 1 of preparative regimen followed by 80 mg daily on each remaining day of the preparative regimen plus 3 additional days + Ondansetron 8 mg orally every 8 h on each day of preparative regimen plus 1 additional day + DEX 7.5 mg IV daily vs. Placebo + Ondansetron 8 mg orally every 8 h on each day of preparative regimen plus 1 additional day + DEX 10 mg IV daily
Bubalo J, et al., 2018 [21]	R, DB, SC	40	Either allo (*n* = 33) Auto (*n* = 17)	Cyclophosphamide conditioning regimens: BuCy (*n* = 23) -Busulfan (BU) PO 16 mg/kg over 4 days + cyclophosphamide (CY) 120 mg/kg over 2 days -Busulfan PO 14 mg/kg over 3.5 days + cyclophosphamide 150 mg/kg over 3 days -Busulfan PO 16 mg/kg over 4 days + cyclophosphamide 200 mg/kg over 4 days CY-TBI (*n* = 17) -TBI 1200 cGy over 4 days in 8 fractions + cyclophosphamide 120 mg/kg over 2 days -TBI 1400 cGy over 4 days in 8 fractions + cyclophosphamide 120 mg/kg over 2 days	AML (*n* = 15) NHL (*n* = 9) MDS (*n* = 4) Myelofibrosis (*n* = 4) ALL (*n* = 3) CML (*n* = 3) CLL (*n* = 1) Waldenstrom’s macroglobulinemia (*n* = 1)	NK1 RA + 5-HT3 RA vs. 5-HT3 RA	APR + OND + DEX (*n* = 20) vs. OND + DEX (*n* = 20)	Aprepitant 125 mg on day 1, then 80 mg until day 4 + Ondansetron 8 mg PO q 6 h × 4 days with BU and 8 mg IV Q12 h × 5 − 9 doses before CY + DEX 20 mg QD × 2–6 days vs. Placebo on day 1 until day 4 + Ondansetron 8 mg PO q 6 h × 4 days with BU and 8 mg IV Q12 h × 5–9 doses before CY + DEX 20 mg QD × 2–6 days
**NK1 RA + 5HT3 RA + DEX + olanzapine vs. NK1 RA + 5-HT3 RA + DEX comparative study**								
Clemmons AB, et al., 2018 [22]	R, DB, SC	101	Subgroup were HSCT Allo (*n* = 24) Auto (*n* = 44)	Conditioning regimen: Melphalan (*n* = 25) BEAM (*n* = 19) BuCy (*n* = 11) FluBu (*n* = 2) FluCy (*n* = 7) FluCy + TBI (*n* = 4) Chemotherapy regimen: 7 + 3 (*n* = 23) ICE (*n* = 10)	AML (*n* = 35) Lymphoma (*n* = 31) MM (*n* = 24) MDS (*n* = 4) CML (*n* = 1) CLL (*n* = 1) ALL (*n* = 3) Other (*n* = 2)	NK1 RA + 5-HT3 RA + DEX + olanzapine vs. NK1 RA + 5-HT3 RA + DEX	FOSAPR + OND + DEX + olanzapine (*n* = 51) vs. FOSAPR + OND + DEX (*n* = 50)	FONDO: Fosaprepitant 150 mg iv on day 1 + Ondansetron 8–16 mg po/iv + DEX 8–20 mg po/iv on each day of CT + Olanzapine 10 mg oral on all CT days plus 3 additional days after CT TBI days: Ondansetron 8 mg po + DEX 4 mg po before each session vs. FOND: Placebo oral on all CT days plus 3 additional days after CT + Fosaprepitant 150 mg iv on day 1 + Ondansetron 8–16 mg po/iv + DEX 8–20 mg po/iv on each day of CT TBI days: Ondansetron 8 mg po + DEX 4 mg po before each session
**NK1 RA regimen single-arm non-comparative studies**								
**Melphalan chemotherapy regimens**								
Loteta B et al., 2020 [23]	SC, OL	106	Autologous	Melphalan 200 mg/m^2^ (*n* = 106)	MM	NK1 RA + 5-HT3 RA	NEPA (no DEX)	Oral NEPA Days 1, 2, and 4
Apolito V et al., 2020 [24]	SC, OL	70	Autologous	High-dose melphalan (*n* = 70)	MM	NK1 RA + 5-HT3 RA + DEX	NEPA + DEX	Oral NEPA + DEX 10 mg PO prior to melphalan
Bechtel T et al., 2014 [25]	SC, OL	26	Autologous	Melphalan 200 mg/m^2^ (*n* = 26)	MM	NK1 RA + 5-HT3 RA + DEX	APR + OND + DEX	Aprepitant 125 mg orally 1 h before HEC on day 2, 80 mg orally 24 and 48 h after initial dose + Ondansetron 16 mg PO day 2 + DEX 12 mg PO day 2, and 8 mg on days 1, 0, and 1
Isoda A et al., 2017 [26]	SC, OL	24	Autologous	Melphalan 100 mg/m^2^ days 1 and 2; ASCT day 4	MM	NK1 RA + 5-HT3 RA + DEX	APR + PALO + DEX	Aprepitant 125 mg on day 1, then 80 mg on days 2–4 + Palonosetron 0.75 mg IV on day 1 + DEX 6.6 mg IV on days 1–4
**Other chemotherapy regimens**								
Jordan K et al., 2011 [27]	SC, OL	64	Autologous	High-dose melphalan (*n* = 21) Age 18–60 years: 100 mg/m^2^: days 1, 2 Age 60–70 years: 70 mg/m^2^: days 1, 2 PBHSCT: day 4 High-dose T-ICE (*n* = 43) Paclitaxel 200 mg/m^2^: day 1 Carboplatin 330 mg/m^2^: days 1–3 Etoposide 330 mg/m^2^: days 1–3 Ifosfamide 3300 mg/m^2^: days 1–3 PBHSCT: day 5	MM (*n* = 21) Solid tumors: Testicular (*n* = 23) Sarcoma (*n* = 15) Thymic carcinoma (*n* = 4) Unknown primary (*n* = 1)	NK1 RA + 5-HT3 RA + DEX	APR + GRAN + DEX	Day 1: Aprepitant 125 mg po + Granisetron 1 mg iv + DEX 8 mg iv Other days of HDC: Aprepitant 80 mg po + Granisetron 1 mg iv + DEX 8 mg iv Days 1, 2 after HDC: Aprepitant 80 mg po + DEX 8 mg iv
Di Renzo N et al., SCC 2022 (chemo-mobilization phase) [28]	MC, OL	81	Autologous	Various with/without ritixumab; all 2–5 days duration Most common: DHAP (*n* = 29) R-DHAP (*n* = 20) IEV (*n* = 16) R-DHAOX (*n* = 5)	NHL	NK1 RA + 5-HT3 RA	NEPA (no DEX)	Oral NEPA Days 1, 3, and 5 (if mobilization lasted 5 days)
Di Renzo N et al. BMT 2020 (Conditioning phase) [29]	MC, OL	70	Autologous	BEAM (*n* = 23) (carmustine 300 mg/m^2^ on day − 6, etoposide 200 mg/m^2^, and cytarabine 400 mg/m^2^ on days − 5, − 4, − 3, and − 2, and melphalan 140 mg/m^2^ on day − 1) FEAM (*n* = 46) (fotemustine 300 mg/m^2^ on day − 6, etoposide 200 mg/m^2^, and cytarabine 400 mg/m^2^ on days − 5, − 4, − 3, and − 2, and melphalan 140 mg/m^2^ on day − 1) Melphalan/mitoxatrone (*n* = 1)	NHL	NK1 RA + 5-HT3 RA	NEPA (no DEX)	Oral NEPA Days − 6, − 4, and − 2
Bubalo JS et al., 2024 [30]	SC, OL	43	Autologous	BEAM (*n* = 43)	HL (*n* = 14) B cell lymphoma (*n* = 13) Peripheral T cell lymphoma (*n* = 9) Mantle cell lymphoma (*n* = 7)	NK1 RA + 5-HT3 RA	NEPA + DEX	Oral NEPA Day − 6, − 4, − 1 DEX 12 mg Day − 6, then 8 mg daily days − 5 to − 1
Paul B et al., 2010 [31]	SC, OL	42	Either autologous (*n* = 39) or allogenic (*n* = 3)	BEAM (*n* = 17) High-dose melphalan (*n* = 14) BuCy (*n* = 4) R-BEAM (*n* = 2) CyTBI (cyclophosphamide and total body irradiation) (*n* = 2) BEAM + BEXXAR (*n* = 1)	MM (*n* = 15) NHL (*n* = 15) HL (*n* = 5) MDS/AML (*n* = 3) APL (*n* = 2) Mantle cell lymphoma (*n* = 2)	NK1 RA + 5-HT3 RA + DEX	APR + OND/DOL + DEX	Aprepitant 125 mg on day 1, then 80 mg on days 2–3 + Ondansetron 24 mg or Dolasetron 100 mg PO on day 1 + DEX 12 mg PO on day 1 For patients receiving CT over multiple days: Aprepitant 80 mg daily throughout CT
Abidi MH et al., 2012 [32]	SC, OL	35	Autologous	High-dose cyclophosphamide and filgrastim (*n* = 40)	MM (*n* = 19) NHL (*n* = 11) HL (*n* = 4) APL (*n* = 1)	NK1 RA + 5-HT3 RA	APR + GRAN + DEX	Aprepitant 125 mg on day 1, then 80 mg on days 2–3 + Granisetron (1 mg oral or 10 mg IV) on day 1 + DEX 10 mg PO on day 1
**5-HT3 RA non-comparative studies**								
**Predominantly melphalan chemotherapy regimens**								
Musso M et al., 2010 [33]	SC, OL	134	Autologous	Multiple myeloma: Melphalan 200 mg/m^2^ (*n* = 33) Melphalan 140 mg/m^2^ (*n* = 19) NHL/HL: BEAM (*n* = 23) carmustine 150 mg/m^2^ on days − 7 and − 6, etoposide 200 mg/m^2^ on days − 5, − 4, − 3, and − 2, cytarabine 400 mg/m^2^ on days − 5, − 4, − 3, and − 2, melphalan 140 mg/m^2^ on day − 1 FEAM (*n* = 40) fotemustine 150 mg/m^2^ on days − 7 and − 6, etoposide 200 mg/m^2^ on days − 5, − 4, − 3, and − 2, cytarabine 400 mg/m^2^ on days − 5, − 4, − 3, and − 2, melphalan 140 mg/m^2^ on day − 1 AML: Ida/Ara-C (*n* = 19) idarubicin 8 mg/m^2^ on days − 5 and − 4, cytarabine 2 g/m^2^ twice/day on days − 5, − 4, − 3, − 2, and − 1	MM (*n* = 52) NHL (*n* = 50) AML (*n* = 19) HL (*n* = 13)	5-HT3 RA	PALO + DEX	Palonosetron 0.25 mg IV + DEX 8 mg IV administered 30 min before starting CT. An additional dose of DEX (4 mg twice daily IV) was administered every other day during the conditioning regimen
LaPorte J et al., 2019 [34]	SC, OL	85	Autologous	Melphalan (*n* = 48) Busulfan/cyclophosphamide/etoposide (*n* = 23) BEAM (*n* = 13) Busulfan/cyclophosphamide (*n* = 1)	MM (*n* = 47) NHL (*n* = 24) HL (*n* = 13) ALL (*n* = 1)	5-HT3 RA	OND + PALO + DEX	Each day of IV CT up to last: Ondansetron 8 mg IV + DEX 10 mg IV + Last or only day of CT: Palononsetron 0.25 mg IV + DEX 10 mg IV + Days 1–2 after CT: DEX 8 mg PO daily
Giralt SA et al., 2011 [35]	R, DB, MC	73	Autologous	Melphalan (*n* = 73) 100 mg/m^2^ on days − 2 and − 1 before HSCT	MM	5-HT3 RA	PALO for 1 day (*N* = 24), 2 days (*N* = 24) or 3 days (*N* = 25)	1 day: Palonosetron 0.25 mg IV on day − 2 2 days: Palonosetron 0.25 mg IV on days − 2 and − 1 3 days: Palonosetron 0.25 mg IV on days − 2, − 1, and 0 DEX 20 mg IV given to all patients on days − 2 and − 1 immediately before or after the study drug
**Other chemotherapy regimens**								
Lopez-Jimenez J, et al., 2006 [36]	MC, OL	177	Either autologous (*n* = 56) or allogenic (*n* = 44)	Transplant group (*n* = 100): BUCY (*n* = 29) BEAM (*n* = 26) CY-TBI (*n* = 26) CBV (*n* = 19) Non-transplant leukemia group (*n* = 77): Standard dose ara-C + anthracycline ± VP-16 (*n* = 46) Intermediate dose ara-C + anthracycline + VP-16 (*n* = 3) High-dose ara-C ± others (*n* = 28)	AML (*n* = 105) ALL (*n* = 11) HL (*n* = 13) NHL (*n* = 26) MM (*n* = 8) CML (*n* = 6) Other (*n* = 8)	5-HT3 RA	Primarily OND (no DEX)	5-HT3 RA (ondansetron primarily used in 95.8% patients) Other anti-emetic drugs, mainly anti-dopaminergics, were added for 27.3% and 28% of patients receiving CT and transplant, respectively No DEX
Martino M et al., 2020 [37]	SC, OL	88	Autologous	Cyclophosphamide 2 g/m^2^	MM	5-HT3 RA	GRAN + DEX	Granisetron transdermal system on day − 2 of CY and day 5 post CY + DEX 8 mg IV on the day of CY infusion
Matsuoka S et al., 2003 [38]	R, SC, OL, AC	50	Either allogenic (*n* = 36) or autologous (*n* = 14)	TBI + HD CT (*n* = 32) TBI + ara-C + CY (*n* = 13) TBI + CY (*n* = 12) TBI + ara-C (*n* = 5) TBI + melphalan (*n* = 2) HD CT (*n* = 18) MCVAC (*n* = 12) BU + CY (*n* = 4) BEAM (*n* = 1) ICE (*n* = 1)	Acute Leukemia (*n* = 17) Lymphoma (*n* = 14) CML (*n* = 11) Other (*n* = 8)	5-HT3 RA	GRAN + DEX vs. GRAN without DEX	Granisetron 40 µg/kg + DEX (4 mg, i.v) 30 min before each dose of CT or TBI (and repeated every 12 h) vs. Granisetron as above without DEX

Study design abbreviations: *AC*, active control; *CO*, crossover; *CT*, chemotherapy; *MC*, multicenter, *OL,* open label; *PC,* placebo-controlled; *R*, randomized; *SB,* single blind; *SC,* single center

Chemotherapy/conditioning regimens abbreviations: *ABVD*, adriamycin + bleomycin + vinblastine + dacarbazine; *Ara-C*, cytarabine; *BEAC*, carmustine + etoposide + cytarabine + cyclophosphamide; *BEAM*, carmustine + etoposide + cytarabine + melphalan; *BCV*, carmustine + etoposide + cyclophosphamide; *Bu/Cy*, busulfan + cyclophosphamide; *CEOP*, cyclophosphamide + epirubicin + vincristine + prednisone; *CHOP*, cyclophosphamide + doxorubicin + vincristine + prednisone; *DHAP*, dexamethasone + high-dose cytarabine + cisplatin; *ECC*, epirubicin + cyclophosphamide; *ESHAP*, etoposide + methylprednisolone + cytarabine + cisplatin; *FEAM*, fotemustine + etoposide + cytarabine + melphalan; *FluBu,* fludarabine + busulfan; *FluCy*, fludarabine + cyclophosphamide; *ICE*, ifosfamide + carboplatin + etoposide; *Ida/Ara-C*, idarubicin + cytarabine; *IVE*, ifosfamide + epirubicin + etoposide; *R-DHAOX*, rituximab + dexamethasone + high-dose cytarabine + oxaliplatin; *MCVAC*, ranimustine + etoposide + cytarabine + cyclophosphamide; *MEAM*, ranimustine + etoposide + cytarabine + melphalan; *ProMACE***-***CytaBOM*, prednisone + doxorubicin + cyclophosphamide + etoposide + cytarabine + bleomycin + vincristine + methotrexate + leucovorin; *R***-***DHAP*, rituximab + dexamethasone + high-dose cytarabine + cisplatin; *R-CHOP*, rituximab + cyclophosphamide + doxorubicin + vincristine + prednisone; *R-CHP*, rituximab + cyclophosphamide + doxorubicin + prednisone; *R-COMP*, rituximab + cyclophosphamide + vincristine + liposomal doxorubicin + prednisone; *R-THPCP*, rituximab + cyclophosphamide + pirarubicin + prednisone; *T-ICE*, paclitaxel + ifosfamide + carboplatin + etoposide; *VCD*, bortezomib + cyclophosphamide + dexamethasone; *TBI*, total body irradiation; *VP-16*, etoposide

Cancer type abbreviations: *ALL*, acute lymphocytic leukemia; *AML*, acute myeloid leukemia; *APL*, acute promyelocytic leukemia; *CML*, chronic myeloid leukemia; *HL*, Hodgkins lymphoma; *MDS*, myelodysplastic syndromes; *MM*, multiple myeloma; *NHL*, non-Hodgkins lymphoma

Antiemetic abbreviations: *APR*, aprepitant; *DEX*, dexamethasone; *FOSAPR*, fosaprepitant; *GRAN*, granisetron; *iv*, intravenous; *NEPA*, netupitant/palonosetron; *NK1*, neurokinin 1; *OND*, ondansetron; *PALO*, palonosetron; *RA*, receptor antagonist

### Study designs and features

The studies identified in this search conducted in the hematology setting are heterogenous, with varying characteristics pertaining to randomization, control groups, size, patient populations, cancer types, chemotherapies, antiemetics, and assessments (Table 1). Of all 22 studies, the majority were single-center (*n* = 18), non-comparative studies (*n* = 17) with small sample sizes (*n* = 16 with < 100 patients). All studies included only adults. One study was in the allogeneic transplant setting, 15 were autologous, and the remaining 6 recruited patients scheduled to receive either type of transplantation.

While the types of malignancies encompassed a broad range, the most common were multiple myeloma, non-Hodgkins lymphoma, and acute leukemia.

As expected in the HSCT setting, the most common conditioning chemotherapy regimens were high-dose melphalan, BEAM (carmustine, etoposide, cytarabine, melphalan), FEAM (fotemustine, etoposide, cytarabine, melphalan), and high-dose cyclophosphamide.

An NK1 RA regimen was evaluated 15 of the 22 studies, most (*n* = 10) of which were non-comparative. The comparative studies (*n* = 4) assessed the benefit of an NK1 RA with a 5-HT3 RA (with/without DEX) over a 5-HT3 RA (with/without DEX) alone, and one study evaluated the benefit of adding olanzapine to an NK1 RA triplet regimen; this study included both transplant and non-transplant patients. The remaining studies (*n* = 7) were non-comparative studies evaluating a 5-HT3 RA with/without DEX.

### Efficacy outcomes

While complete response (CR), defined as no emesis and no use of rescue medication, has been the gold standard clinical endpoint for years in antiemetic trials, no such standard exists for assessing antiemetic efficacy in the hematology setting.

In the studies identified in this search, there was no consistency in the primary efficacy endpoint (Table 2). Approximately half (*n* = 12) of the studies designated CR, as traditionally defined, as the primary endpoint. Some studies used the terminology of “complete response” but defined it differently. Alternative definitions of CR included (1) no emesis and less than grades 1–2 nausea; (2) no more than one emetic episode; (3) no emesis and no mild/moderate nausea; (4) no emesis, no nausea, and no rescue use; and (5) no emesis and no more than mild nausea. Additional primary efficacy endpoints included “complete protection” defined as no vomiting only; “complete emetic response” defined as no emesis, none to mild nausea, and no rescue use; and “complete control” defined as no emesis.

**Table 2:** Efficacy outcomes for antiemetics in patients receiving conditioning for stem cell transplantation

Study	HSCT type	Antiemetics/study arms	DEX	Primary/key efficacy endpoint(s) definition	Nausea assessment	Primary phase(s) assessed/definition	Complete response	No nausea	No emesis
**Allogeneic**									
Yeh SP et al. 2014 [17]	Allogenic	Palonosetron + DEX	Yes	Not defined; Assessed no vomiting (CP) and no nausea	100-mm VAS scale (0 = no nausea; 100 = as bad as it can be)	1) Early: conditioning period 2) Late: first week after HSCT (days 0–7)	NR	Early: 22.2% Late: 11.1%	Early: 37% Late: 37%
**Autologous or both**									
**NK1 RA + 5HT3 RA + DEX vs. 5-HT3 RA + DEX comparative studies**									
**Predominantly melphalan chemotherapy regimens**									
Schmitt T et al., 2014 [18]	Autologous	Aprepitant + granisetron + DEX vs. Placebo + granisetron + DEX	Yes	CR: no emesis and no rescue medication	100-mm VAS (< 5 = no nausea)	Overall: 0–120 h after melphalan	58% APR vs. 41% GRAN (*p* = 0.0042)	85% APR vs. 78% GRAN (*p* = 0.106)	78% APR vs. 65% GRAN (*p* = 0.0036)
Svanberg A et al., 2015 [19]	Autologous	Aprepitant + tropisetron + betamethasone vs. placebo + tropisetron + betamethasone	Yes	CR: no emesis, no nausea, and no use of rescue medication	VAS scale 0–10	Acute: during CT Delayed: up to 10 days after the end of HDCT	Primary endpoint not reported	No significant difference	83% APR vs. 36% 5-HT3 RA (*p* = 0001) (from baseline to day 10)
**Cyclophosphamide chemotherapy regimens**									
Stiff PJ et al., 2013 [20]	Autologous and allogenic	Aprepitant + ondansetron + DEX vs. placebo + ondansetron + DEX	Yes	CR: no emesis with only grades 1–2 nausea	100-mm VAS (0 = no nausea)	Composite: average daily response during 5–8 days of HCT	81.9% APR vs. 65.8% OND (*p* < 0.001)	NR	73.3% APR vs. 22.5% OND (*p* < 0.001)
Bubalo J, et al., 2018 [21]	Autologous and allogenic	Aprepitant + ondansetron vs. placebo + ondansetron	Yes	CR: no emesis and the absence of mild to moderate nausea	100-mm VAS (6–70 mm = mild to moderate)	From first dose of CT to 7 days after HSCT	40% APR vs. 20% OND	Reported only daily (more APR patients had no nausea on days 1–3 and 14–15)	Not reported separately from CR
**NK1 RA + 5HT3 RA + DEX + olanzapine vs. NK1 RA + 5-HT3 RA + DEX comparative study**									
Clemmons AB, et al., 2018 [22]	Subset of autologous and allogenic	Fosaprepitant (F) + ondansetron (ON) + DEX (D) + olanzapine (O) (FONDO) vs. FOND	Yes	CR: No emesis and no more than minimal nausea (< 25 mm 100-mm VAS)	100-mm VAS (< 5 = none; < 25 = minimal)	Acute: CT days Delayed: 5 days after CT Overall: CT days + 5 days after	Overall phase for all patients: 55% FONDO vs. 26% FOND (*p* = 0.003)	Overall phase for all patients:: 39% FONDO vs. 13% FOND (*p* = 0.006)	Not reported separately from CR
**NK1 RA regimen single-arm non-comparative studies**									
**Melphalan chemotherapy regimens**									
Loteta B et al., 2020 [23]	Autologous	NEPA	No	CR: no emesis and no use of rescue medication (during the overall phase)	Graded per CTCAE 4.03	Acute: 0–24 h Delayed: 25–120 h Overall: days 1–5	Overall: 93.3% Acute: 94.3% Delayed: 95.2%	NR	Overall: 93.4% Acute: 94.3% Delayed: 96.2%
Apolito V et al., 2020 [24]	Autologous	NEPA + DEX	Yes	CR: no emesis and no use of rescue medication (during the overall phase)	100-mm VAS (< 5 = none; > 25 = significant)	Acute: 0–24 h Delayed: 25–120 h Overall: 0–120 h after chemotherapy	Overall: 56% Acute: 83%	Overall: 39% Acute: 78%	Overall: 85% Acute: 98%
Bechtel T et al., 2014 [25]	Autologous	Aprepitant + Ondansetron + DEX	Yes	CR: defined as no more than 1 emetic episode (during the delayed phase)	Linear analog scale 0–10	Delayed: 24–120 h after CT	Delayed: 96.2%	Delayed: 11.5%	Delayed: 92.3%
Isoda A et al., 2016 [26]	Autologous	Aprepitant + palonosetron + DEX	Yes	CR: No emesis and no use of rescue medication	4-point Likert scale (0 = none; 3 = severe)	Primary: Overall: 0–120 h after completion of melphalan Secondary: Acute: 0–48 h Delayed: 48–120 h Extended: 120–168 h	Overall: 75% Acute: 88% Delayed: 75% Extended: 67%	Reported daily time course of nausea severity	Overall: 92% Acute: 92% Delayed: 100%
**Other chemotherapy regimens**									
Jordan K et al., 2011 [27]	Autologous	Aprepitant + granisetron + DEX	Yes	CR: no emesis and no use of rescue medication	Not reported	Acute: during days of CT Delayed: day 1 until 5 days after end of CT Overall: day 1 until 5 days after end of CT	Overall: 63% Acute: 83% Delayed: 70% (response rates higher for T-ICE than melphalan)	Overall: 53% Acute: 80% Delayed: 76%	NR
Di Renzo N et al., 2022 [28]	Autologous (mobilization phase)	NEPA	No	CR: no emesis and no use of rescue medication (during the overall phase)	4-point Likert scale (0 = none; 3 = severe)	Acute: day 1 to last day of CT Delayed: last day of CT until 48 h after last dose of CT Overall: from day 1 until 48 h after last dose of CT	Overall: 77.8% Acute: 90.1% Delayed: 84.0%	Overall: 39% Acute: 56% Delayed: 54%	Overall: 86.4% Acute: 93.8% Delayed: 90.1%
Di Renzo N et al., 2020 [29]	Autologous (conditioning phase)	NEPA	No	CR: no emesis and no use of rescue medication (during the overall phase)	4-point Likert scale (0 = none; 3 = severe)	Acute: days 1–6 (during CT) Delayed: days 7–8 Overall: days 0–8	Overall: 87.1% Acute: 88.6% Delayed: 98.6%	Overall: 40% Acute: ~ 43% Delayed: ~ 63%	Overall: 88.6% Acute: 90.0% Delayed: 98.6%
Bubalo JS et al., 2024 [30]	Autologous	NEPA	Yes	CR: no emesis, no more than mild to moderate nausea, and no use of rescue medication	10-point scale (< 1 = none; 1–3 = mild; 4–6 = moderate; 7–10 = severe)	Acute: during 6 days of CT (0–144 h) Delayed: through 5 days after end of CT (145–264 h) Overall: entire 11-day period (0–264 h)	Overall: 30.9%	NR	Acute: 100% Delayed: 81%
Paul B et al., 2010 [31]	Autologous & Allogenic	Aprepitant + ondansetron or dolasetron + DEX	Yes	Complete Emetic Response (CER): no episodes of emesis, none to mild nausea, and no breakthrough medication use	100-mm VAS (0–30 = none to mild; 31–60 = moderate; ≥ 61 = severe)	Days 1–7	Average days 1–7: 54%; range from 42.9 to 73.8% across each day	None to mild ranged from 27 to 37% each day on days 1–7	No emesis rates ranged from 32 to 41% each day on days 1–7
Abidi MH et al., 2012 [32]	Autologous	Aprepitant + granisetron + DEX	Yes	CR: no emesis and no use of rescue medication (during the acute phase)	100-mm VAS (0 = none; 5–25 = mild; > 25 = severe)	Acute: 0–24 h Delayed: 25–120 h Overall: days 1–5	Acute: 57% Delayed and Overall: NR	Overall: 43% no more than mild nausea	Delayed: 63%
**5-HT3 RA non-comparative studies**									
**Predominantly melphalan chemotherapy regimens**									
Musso M et al., 2010 [33]	Autologous	Palonosetron + DEX	Yes	CR: no emesis and no use of rescue medication	CTCAE v.3	Overall: throughout the conditioning regimen and up to 5 days after the end of the conditioning regimen	Overall: 36% (highest with Ida/Ara-C, lowest with melphalan 200 mg/m^2^ conditioning)	Overall: 35%	Overall: 48%
LaPorte J et al., 2018 [34]	Autologous	Ondansetron + DEX prior to CT, then palonosetron + DEX on last day of CT	Yes	CR: No emetic episodes and no use of rescue medication (during the delayed phase)	10-point Likert scale (1 = none; 2–4 = mild; 5–7 = moderate; 8–10 = severe)	Acute: 0–24 h after CT Delayed: 24–120 h after CT Overall: 0–120 h after CT	Delayed: 15% Acute: 39% Overall: 12%	NR	52%
Giralt SA et al., 2011 [35]	Autologous	Palonosetron (1 day) + DEX vs. palonosetron (2 days) + DEX vs. palonosetron (3 days) + DEX	Yes	CP: defined as the proportion of patients with no emetic episodes throughout the cumulative 7-day study period (days − 2 through + 4)	4-point Likert scale (0 = none; 3 = severe)	Overall: days − 2 to day + 4	PALO 1 day: 8% PALO 2 days: 21% PALO 3 days: 20%	PALO 1 day: 8% PALO 2 days: 29% PALO 3 days: 16%	PALO 1 day: 42% PALO 2 days: 42% PALO 3 days: 44%
**Other chemotherapy regimens**									
Lopez-Jimenez J, et al., 2006 [36]	Subset of autologous and allogenic	5-HT3 RA (primarily ondansetron) (+ anti-dopaminergics in 28% patients)	No	CR: no emesis and no use of rescue medication	100-mm VAS scale (0 = no nausea, 100 = as bad as it can be)	Acute: 0–24 h Delayed: 25–120 h Overall: days 1–5	HSCT subset: Acute: 69% Delayed: 21% Overall: 19%	HSCT subset: Acute: 49% Delayed: 13% Overall: 13%	HSCT subset: Acute: 69% Delayed: 22% Overall: 20%
Martino M et al., 2020 [37]	Autologous	Granisetron transdermal + DEX	Yes	CR: no emesis and no use of rescue medication	CTCAE	Overall: during and for 15 days after CT admin	Extended overall: 46%	Extended overall: 34%	Extended overall: 46%
Matsuoka S et al., 2003 [38]	Autologous and allogenic	Granisetron + DEX vs. granisetron	Yes	CC: no vomiting episodes MC: 1–2 vomiting episodes NC: 3 + vomiting episodes	Not reported	Each 24 h, calculated as % of patient days during conditioning	NR	NR	CC (% of patient days): GRAN: 76% GRAN + DEX: 70%

Shaded cells means standard complete response (CR) definition (i.e., no emesis and no use of rescue medication) and standard evaluation phase. *CC*, complete control; *CER*, complete emetic response; *CP*, complete protection; *CT*, chemotherapy; *CTCAE*, common terminology criteria for adverse events; *DEX*, dexamethasone; *FOND-O*, fosaprepitant, ondansetron, dexamethasone + olanzapine; *FOND*, fosaprepitant, ondansetron + dexamethasone; *h*, hours; *HSCT*, hematopoietic stem cell transplantation; *MC*, major control; *NC*, no control; *NR*, not reported; *PC*, partial control; *PR*, partial response; *VAS*, visual analog scale

Similarly, there was variability in the tool used to assess the subjective endpoint of nausea, with a visual analog scale being used in about half of the studies and a Likert scale (of either 4-point or 10-point) being used in about a quarter of the studies. A few trials graded nausea based on the common terminology criteria for adverse events (CTCAE) (Table 2).

As the doses, schedules, and emetic time course of the various chemotherapy regimens vary, it is difficult to set a consistent timeframe for assessing antiemetic efficacy. In the solid tumor setting where chemotherapy is generally administered as a single dose, the acute (0–24 h), delayed (> 24–120 h), and overall (0–120 h) phases post-chemotherapy have been consistently utilized in assessing antiemetic efficacy. Not surprisingly, in these hematology studies, this consistency was not seen. Ten of the studies assessed antiemetic efficacy from 0 to 120 h or up to 5 days post-chemotherapy while the remaining assessed efficacy during the acute phase, or up to days 4 or 7 or 8 or in one case, day 15.

Table 2 summarizes the efficacy outcomes as reported in these HSCT trials.

Cross-comparison of outcomes is difficult due to the varied conditioning regimens, different patient populations/cancer types, antiemetics, endpoints, and timepoints for assessment.

In the studies that included an NK1 RA regimen, the results encompassed a broad range. Complete response rates (by the standard definition only) ranged from 56 to 93%, with a range of 58 to 75% for aprepitant regimens and 56 to 93% for NEPA (netupitant/palonosetron). Not surprisingly, across all NK1 RA inclusive studies regardless of the complete response definition, the range of CR rates was wider, from 26 to 96% for the NK1 RA regimens.

In the three NK1 RA studies that included a 5-HT3 RA comparator, the complete response (standard definition) rates for the 5-HT3 RA arm were significantly lower than the NK1 RA arm with a range of 20 to 66%. The CR rate for the 5-HT3 RA (granisetron) arm in the only study utilizing the standard definition of CR was 41%.

In the few studies that included only a 5-HT3 RA, the CR rates (by the standard definition only) ranged from 12 to 36%.

The studies inconsistently reported emesis and nausea control; however, in those studies that reported both endpoints, no nausea rates tended to be lower than no emesis rates.

## Discussion

In the absence of clear guidance from international antiemetic guidelines on the optimal prophylactic antiemetic regimens and schedules for patients receiving various high-dose chemotherapy regimens for hematologic malignancies, this comprehensive review sought to explore the evidence in the literature.

While drawing definitive conclusions is difficult in this setting, the following were our key observations. Numerous studies appear to have underutilized antiemetics that some guidelines suggest should be used (i.e., NK1 RAs and olanzapine). Fifteen of the 22 studies (68%) in this literature review included an NK1 RA regimen; the remainder focused on a 5-HT3 RA (with/without DEX) only. Only a few studies (*n* = 5) included NEPA, the newest NK1 RA/5-HT3 RA fixed combination antiemetic that has a longer half-life and extended receptor occupancy compared with aprepitant and offers some evidence of protection from nausea and vomiting for a longer duration [39–42]. NEPA may be particularly useful in this setting where chemotherapy may be given for a longer duration and where delayed CINV is a significant concern.

In clinical practice, the use of NK1 RA-containing regimens is likely even lower than that seen across these clinical trials. In the survey of the hematologists/oncologists in Europe [16], only 36% of respondents indicated that an NK1 RA/5-HT3 RA/steroid combination was being administered on day 1 to prevent CINV in their patients receiving a highly emetogenic conditioning regimen prior to HSCT; benzodiazepines, other neuroleptics, metoclopramide, and olanzapine were the most commonly administered agents after day 1.

The comparative HSCT studies in this review that evaluated an NK1 RA regimen vs. a 5-HT3 RA showed significantly better CINV control with the NK1 RA regimen (regardless of the efficacy endpoint evaluated). Consistent with this, in the studies where only a 5-HT3 RA was administered (with/without DEX), the response rates were generally lower than in the studies where an NK1 RA was given. In the one study that added olanzapine to the NK1 RA triplet, CINV control was better in the olanzapine arm than the NK1 RA arm [22]. This data reinforces the available guidance and validates utilization of an NK1 RA-containing regimen as standard prophylaxis in the HSCT setting. Additional studies are needed with olanzapine as adding it to the regimen may offer further benefit [43], particularly in patients with elevated emetic risk or in those prone to experiencing nausea. However, little guidance exists as to the optimal strategy for incorporating olanzapine-based regimens into HSCT [43]. MASCC/ESMO recently updated their guidance [15] not only to reinforce that an NK1 RA-containing regimen should be used in patients receiving high-dose chemotherapy for HSCT (in their case based on two studies [18, 20]), but they also indicate that olanzapine could be considered as part of the antiemetic regimen. Both MASCC/ESMO and NCCN [10, 15] suggest that if olanzapine is used, it should be administered at bedtime on day 1 and continued for 2–3 days after chemotherapy as these regimens are likely to cause significant delayed emesis.

The range of response rates was wide with numerous studies showing complete response, no emesis or no nausea rates of less than 50%. These reflect a significant clinical problem in preventing CINV in patients with hematologic malignancies. However, it is noteworthy that there are confounding factors that make clinical research in this area extremely difficult. Nausea and vomiting may occur due to other causes in addition to CINV, including prophylactic antibiotics and opioids, for example, that are used for treatment of painful mucositis. The use of total-body irradiation can also be a confounding factor, as many patients will or have experienced emesis with prior chemotherapy or irradiation.

There is a wide variety of high-dose chemotherapy regimens administered over consecutive days, and the time course of emesis/nausea is not well understood. This makes the antiemetic scheduling difficult and also complicates determination of the antiemetic efficacy assessment phase.

Notably, this review is subject to several important limitations that constrain the generalizability and strength of its conclusions. First, there is significant heterogeneity in the studies regarding the chemotherapy/conditioning regimens, antiemetic regimens, study designs, and patient populations. Second, antiemetic strategies varied widely, with many regimens falling short of current guideline recommendations, making it difficult to assess the true efficacy of optimal antiemetic prophylaxis. Third, the majority of studies were non-randomized single-arm studies. The lack of well-powered randomized controlled trials in this population is a critical gap in the literature. Lastly, inconsistent use of antiemetic efficacy endpoints hinders cross-study comparisons and interpretation of outcomes. In contrast to the solid tumor setting where standardized antiemetic endpoints are well-defined and widely adopted, hematology studies often lack this methodological consistency. This represents a critical barrier to evidence synthesis and underscores the urgent need for consensus on standardized endpoints specific to this population. These limitations underscore the need for well-designed, multicenter, randomized trials using standardized outcomes to guide evidence-based antiemetic prophylaxis in this HSCT setting. Future research should also explore additional factors that may influence antiemetic efficacy, including the potential for drug-drug interactions associated with intensive conditioning regimens, and the impact of effective CINV control on nutritional status and overall clinical outcomes in HSCT recipients.

Our findings are consistent with prior reviews which have explored antiemetic efficacy in this setting [44–47]. The earliest review by Tendas and colleagues [45] in 2019 narrowly focuses on patients with multiple myeloma receiving high-dose melphalan; the Yuda review [44] is more guideline-based; the Acuri review [47] includes randomized controlled trials with a focus on trial design; and the Baez-Gutierrez review [46] is the most comprehensive and up to date. Our review, in contrast to these is broader and generally includes the studies referenced in these other reviews. Despite these differences in scope and methodology, all of these reviews, consistent with ours, highlight the need for tailored antiemetic strategies, the complexity of managing CINV in this high-risk population, and acknowledge that guideline-based prophylaxis is inconsistently applied across studies and clinical practice.

## Conclusions

In conclusion, the results of this literature review underscore the pressing need for more robust randomized trials in the hematology and HSCT setting while acknowledging the complexity of determining the emetogenicity of the various chemotherapy regimens and the optimal antiemetic strategies. The scarcity of studies and the heterogeneity of the study features highlight the challenges inherent in this area. However, further research is necessary to improve the overall well-being and quality of life of patients undergoing hematological malignancy treatments.

## Data Availability

No datasets were generated or analysed during the current study.
